# First-trimester calculated remnant cholesterol and gestational diabetes mellitus in twin pregnancies: a retrospective cohort study

**DOI:** 10.3389/fendo.2026.1882821

**Published:** 2026-09-11

**Authors:** Kejun Ye, Yixin Chen, Lin Lin, Ruru Jia, Chen Yingjie, Mengjia Peng

**Affiliations:** 1 Department of Obstetrics and Gynecology, Laboratory for the Diagnosis and Prevention of Diabetic Complications, The Third Affiliated Hospital of Wenzhou Medical University, Ruian, Zhejiang, China; 2 College of Medicine, Zhejiang University, Hangzhou, Zhejiang, China; 3 Department of Emergency, Shishi Municipal Hospital, Quanzhou, Fujian, China

**Keywords:** calculated remnant cholesterol, chorionicity, gestational diabetes mellitus, lipid metabolism, twin pregnancy

## Abstract

**Background:**

Gestational diabetes mellitus (GDM) is particularly prevalent in twin pregnancies, yet the underlying metabolic pathways remain incompletely elucidated. Remnant cholesterol (RC), derived from standard lipid measurements, has been associated with glucose metabolic disorders, but its association with GDM in twin pregnancies is unknown. Whether chorionicity modifies this association has not been investigated.

**Methods:**

This retrospective cohort study included 347 women with twin pregnancies who delivered at a tertiary hospital in China between 2020 and 2025. First-trimester calculated RC was derived as total cholesterol minus LDL-C and HDL-C. Its association with GDM was examined using multivariable logistic regression and restricted cubic splines. Potential effect modification by chorionicity was evaluated using interaction analysis. As a secondary exploratory analysis, the stand-alone discrimination of calculated RC was assessed using ROC curve analysis, and a data-derived cut-off was identified using the Youden index.

**Results:**

After adjustment for age, education, pre-pregnancy body mass index, assisted reproductive technology, gravidity, primiparous status, and chorionicity, calculated RC was associated with GDM (*P* for overall=0.024), with no evidence of non-linearity (*P* = 0.673). The internally derived exploratory cut-off was 1.145 mmol/L, with an AUC of 0.649 (95% CI 0.572–0.724), specificity of 44.95%, and positive predictive value of 22.93%. Calculated RC ≥1.145 mmol/L was associated with higher odds of GDM (adjusted OR 2.56, 95% CI 1.28–5.10; *P* = 0.008). An exploratory interaction by chorionicity was observed (*P* for interaction=0.024), but the monochorionic subgroup contained only 10 GDM events and the estimate was imprecise.

**Conclusions:**

Elevated first-trimester calculated RC was associated with subsequent GDM in women with twin pregnancies. The 1.145 mmol/L cut-off was exploratory and internally derived and requires external validation, whereas the potential heterogeneity by chorionicity should be regarded as hypothesis-generating and further explored in larger prospective cohorts.

## Graphical Abstract

**Figure:**
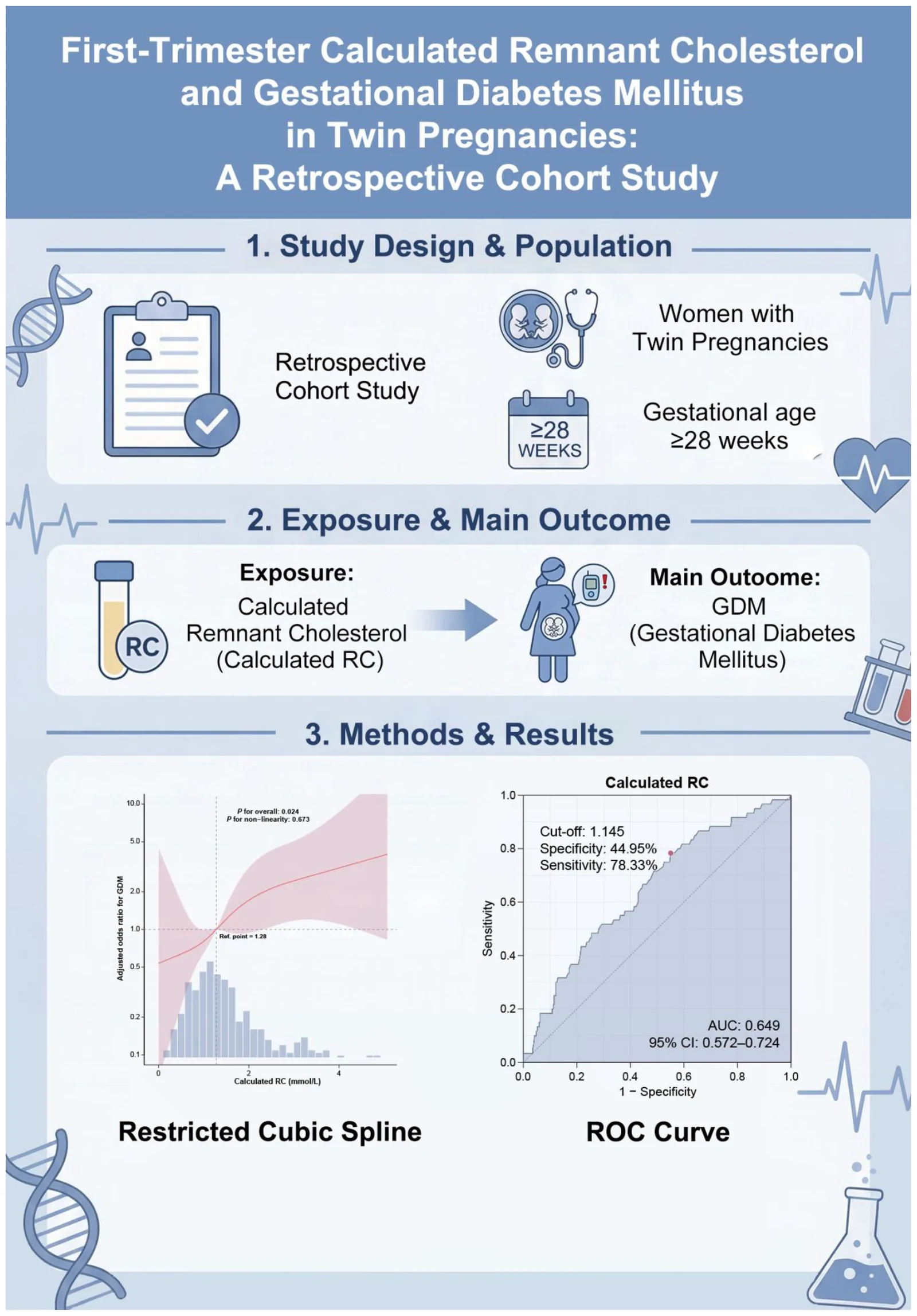

## 1 Introduction

Gestational diabetes mellitus (GDM) is one of the most common metabolic complications of pregnancy. According to estimates from the International Diabetes Federation (IDF), approximately 14% of pregnancies worldwide are affected by GDM, with the Southeast Asia and Western Pacific regions bearing a particularly heavy burden (1). GDM is not only closely associated with adverse perinatal outcomes including macrosomia, birth trauma, and neonatal hypoglycemia (2), but also exerts a profound impact on long-term maternal metabolic health (3). In recent years, the rising prevalence of pre-pregnancy overweight/obesity and the widespread application of assisted reproductive technology (ART) have further amplified the disease burden of GDM (4, 5), making early identification of high-risk populations and primary prevention an urgent priority in perinatal medicine. Against this backdrop, twin pregnancies warrant special attention due to their unique metabolic characteristics. Compared with singleton pregnancies, twin pregnancies exhibit a significantly higher incidence of GDM, with some of this difference being independent of pre-pregnancy body mass index (BMI) (6). The underlying mechanisms are believed to be related not only to the greater total placental hormone secretion and higher degree of insulin resistance in twin pregnancies, but also potentially to early-pregnancy lipid metabolic disturbances (7). However, the specific metabolic pathways underlying the elevated GDM risk in twin pregnancies remain incompletely elucidated, and early metabolic markers associated with GDM in this population remain insufficiently characterized.

Remnant cholesterol (RC) represents the cholesterol content of triglyceride-rich lipoprotein remnants formed after partial hydrolysis by lipoprotein lipase (8). Previous studies have linked elevated RC levels to ischemic heart disease and other cardiometabolic disorders (9). Recent Mendelian randomization studies have also suggested potential causal associations between genetically predicted RC levels and several cardiometabolic outcomes, including type 2 diabetes mellitus (10). These findings raise the possibility that RC may be relevant to disturbances in glucose metabolism, although the biological pathways underlying its association with GDM remain uncertain (11). Evidence regarding RC and GDM is currently limited and has been derived predominantly from singleton pregnancies (12). Whether this association also exists in twin pregnancies remains unclear. Twin pregnancies are characterized by a greater lipid metabolic burden than singleton pregnancies, including an earlier onset and greater magnitude of physiological hyperlipidemia; consequently, RC levels and their association with GDM may differ in this population (13). Chorionicity may contribute further heterogeneity because dichorionic twins have separate placental circulations, whereas monochorionic twins share a placenta and may have intertwin vascular connections and unequal placental sharing (14, 15). Whether these structural differences modify the association between RC and GDM is unknown.

Therefore, the primary objective of this study was to evaluate the association between first-trimester calculated RC and subsequent GDM in women with twin pregnancies, including the shape of the association and potential heterogeneity by chorionicity. A secondary exploratory objective was to characterize the stand-alone discrimination of calculated RC and identify a data-derived cut-off for descriptive purposes. The findings may provide hypothesis-generating evidence for external validation and for future evaluation of whether calculated RC adds information beyond established clinical factors in larger cohorts of women with twin pregnancies.

## 2 Materials and methods

### 2.1 Study design and participants

This was a retrospective cohort study. The study population comprised women with twin pregnancies who delivered at the Department of Obstetrics, Ruian People’s Hospital, Zhejiang Province, China, between 2020 and 2025. This study was approved by the Ethics Committee of Ruian People’s Hospital (approval No. YJ2026021), which waived the requirement for informed consent. Inclusion criteria were: 1) age >18 years; 2) twin pregnancy confirmed by ultrasound; and 3) gestational age at delivery ≥28 weeks. Exclusion criteria were: 1) initial triplet or higher-order pregnancies reduced to twin pregnancies through spontaneous or medical fetal reduction; 2) initial twin pregnancies with one twin lost before 28 weeks of gestation; 3) missing key study variables (e.g., total cholesterol [TC], high-density lipoprotein cholesterol [HDL-C], or low-density lipoprotein cholesterol [LDL-C]) or missing outcome variables precluding covariate adjustment; 4) indeterminate GDM status due to missing clinical information required for guideline-based diagnosis, such as OGTT results; and 5) pre-existing diabetes identified from the medical history documented at the initial antenatal visit, including previous diabetes diagnoses and use of glucose-lowering medication, or overt diabetes detected in early pregnancy, defined as fasting plasma glucose ≥7.0 mmol/L.

### 2.2 Data collection and definitions

#### 2.2.1 Exposure and covariates

Demographic characteristics and clinical data were collected through the electronic medical record system, including age, education level, gravidity, primiparous status, ART, chorionicity, and gestational age at delivery. Anthropometric measurements included height and pre-pregnancy weight. Pre-pregnancy BMI was calculated as weight (kg) divided by height (m) squared. Education level was categorized into three groups: junior high school or below, high school/technical secondary school/vocational high school, and college degree or above. Gravidity was defined as the total number of pregnancies, including the current pregnancy, regardless of pregnancy outcome. Primiparous status was defined as having no previous delivery before the current pregnancy. The current twin fetuses were not counted as previous deliveries. Chorionicity was classified into two groups: dichorionic (DC) twins and monochorionic (MC) twins; the latter included monochorionic diamniotic (MCDA) and monochorionic monoamniotic (MCMA) twins. First-trimester lipid measurements were obtained at 10–14 weeks of gestation from blood collected in the early morning after participants reported being fasting. Because of the retrospective design, the exact fasting duration was not consistently documented and could not be determined. TC, HDL-C, LDL-C, and TG were measured from the same blood sample using a Siemens CH930 automated biochemical analyzer. TG was measured using the glycerol phosphate oxidase–peroxidase method, TC using an enzymatic method, and HDL-C and LDL-C using direct homogeneous assays. All assays were performed in the hospital’s central clinical laboratory under routine calibration and internal quality-control procedures according to the manufacturer’s instructions and the laboratory’s standard operating procedures. Calculated RC was derived as TC − LDL-C − HDL-C, with all components expressed in mmol/L (16). The calculation used the original laboratory-reported values extracted from the electronic medical records, which were reported to two decimal places; no additional rounding was applied by the investigators before calculation. Calculated RC values were independently recomputed from the three lipid components and screened for negative or clearly implausible values. No negative calculated RC values occurred, and no values were truncated or set to zero. Extreme lipid values, including the maximum TG and calculated RC concentrations, were additionally checked against the source laboratory records to verify the recorded values and units and to exclude data-entry, decimal-point, or unit-conversion errors.

#### 2.2.2 Outcome definitions

The primary outcome was GDM, diagnosed by a standard 75-g oral glucose tolerance test (OGTT) performed at 24–28 weeks of gestation according to the International Association of Diabetes and Pregnancy Study Groups (IADPSG) and World Health Organization (WHO) criteria (17, 18). All participants included in the final analytic cohort underwent the same standard 75-g OGTT. The diagnostic thresholds were fasting plasma glucose ≥5.1 mmol/L, 1-hour plasma glucose ≥10.0 mmol/L, or 2-hour plasma glucose ≥8.5 mmol/L, with any one abnormal value sufficient for diagnosis. The fasting, 1-hour, and 2-hour glucose values were extracted directly from the electronic medical record system and manually verified against the original laboratory records. Pre-existing diabetes and overt diabetes detected in early pregnancy were distinguished from GDM. Secondary outcomes included hypertensive disorders of pregnancy (HDP), prelabor rupture of membranes (PROM), gestational age at delivery, and preterm birth (PTB). HDP was defined according to international guidelines from the International Society for the Study of Hypertension in Pregnancy (ISSHP) and the American College of Obstetricians and Gynecologists (ACOG), and included gestational hypertension, preeclampsia, and eclampsia (19, 20). Prelabor rupture of membranes (PROM) was defined as spontaneous rupture of membranes before the onset of labor at any gestational age (21). Gestational age at delivery was calculated from the first day of the last menstrual period to the date of delivery. PTB was defined as delivery occurring at <37 weeks of gestation.

### 2.3 Statistical analysis

The variables included in the reported analyses were complete within the final analytic cohort. No pre-existing statistical power estimates were obtained because the sample size was determined based solely on the available data. All analyses were performed using R software (version 4.2.2; R Foundation for Statistical Computing, Vienna, Austria) and the Free Statistics analysis platform (version 2.4; Beijing, China), an R-based auxiliary analysis interface. Logistic regression models were fitted using the stats package, restricted cubic spline analyses were performed using the rms package, ROC analyses were conducted using pROC, and figures were generated using ggplot2. A two-sided *P* value <0.05 was considered statistically significant. The distributions of continuous variables were evaluated using histograms, quantile–quantile plots, summary measures, and consideration of clinical interpretability. Approximately symmetrically distributed variables were summarized as mean ± standard deviation and compared using the independent-samples Student’s t-test. Skewed continuous variables were summarized as median (interquartile range) and compared using the Mann–Whitney U test. Categorical variables were summarized as frequencies and percentages and compared using the chi-square test or Fisher’s exact test, as appropriate. Baseline comparisons and their *P* values were descriptive and were not used to select covariates for multivariable adjustment.

Subsequently, to explore the shape of the association between calculated RC and GDM, restricted cubic spline (RCS) models based on multivariable logistic regression were constructed. Following the recommendations of Frank et al., knot positions were set at the 5th, 35th, 65th, and 95th percentiles of the calculated RC distribution, and the overall and non-linear associations were assessed using Wald chi-square tests (22). Based on prior literature and clinical expertise, and considering the number of outcome events in this study, multicollinearity was assessed using variance inflation factors (VIFs), with VIF ≥5 indicating substantial multicollinearity. Because of the potential collinearity between TG and calculated RC, TG was not included in the final RCS models. The full-cohort RCS models were adjusted for age, education, pre-pregnancy BMI, ART, gravidity, primiparous status, and chorionicity. In a chorionicity-stratified RCS model, chorionicity was omitted from the adjustment set. Covariates were selected *a priori* on the basis of previous literature and clinical considerations rather than univariable statistical significance (23). The primary continuous and binary calculated RC logistic regression models each contained nine non-intercept parameters. The four-knot RCS term contributed three degrees of freedom, resulting in 11 non-intercept parameters in the full-cohort RCS model. The full-cohort interaction models contained 10 non-intercept parameters, whereas the subgroup-specific binary models contained eight non-intercept parameters after omission of the variable defining the subgroup, and the exploratory RCS model fitted in the dichorionic subgroup contained 10 non-intercept parameters.

As a secondary exploratory analysis, the stand-alone discrimination of calculated RC for GDM was summarized using the ROC curve and AUC. A data-derived cut-off was identified by maximizing the Youden index, and sensitivity, specificity, PPV, and NPV were calculated with Wilson 95% confidence interval (CI) (24). To estimate the uncertainty of the apparent AUC, 2,000 bootstrap samples of the same size as the original cohort were generated by sampling participants with replacement. The AUC was calculated in each bootstrap sample, and the 2.5th and 97.5th percentiles of the bootstrap distribution were used as the 95% CI. This bootstrap procedure was used only to estimate the percentile confidence interval of the apparent AUC and did not constitute optimism correction or out-of-bag validation.

The primary association analysis was conducted using multivariable logistic regression with calculated RC modeled as a continuous linear term. In a secondary exploratory analysis, calculated RC was also analyzed as a binary variable using the ROC-derived cut-off, with the group below the cut-off serving as the reference category. The adjusted model included calculated RC, age, education, pre-pregnancy BMI, ART, gravidity, primiparous status, and chorionicity. In the continuous analysis, the effect estimate represented the change in the odds of GDM per 1 mmol/L increase in calculated RC. Effect estimates were expressed as adjusted odds ratios with 95% CI.

To explore potential heterogeneity in the association between calculated RC and GDM, calculated RC was dichotomized using the ROC-derived cut-off, and three potential effect modifiers were evaluated: age (<35 vs. ≥35 years), ART (no vs. yes), and chorionicity (dichorionic vs. monochorionic). Within each subgroup, the reported OR compared the group above the calculated RC cut-off with the group below the cut-off, with the latter serving as the reference category. Separate multivariable logistic regression models were fitted within each subgroup, excluding the variable defining that subgroup from the corresponding adjusted model. Because of the small number of GDM events in the monochorionic subgroup, the within-stratum estimate for this subgroup was summarized using the crude OR with its 95% CI, and the corresponding *P* value was obtained using Fisher’s exact test. No four-category joint calculated RC–chorionicity exposure was used. Interactions were evaluated by adding cross-product terms between binary calculated RC and each potential effect modifier to full-cohort multivariable logistic regression models and comparing models with and without the interaction term using likelihood ratio tests. Interaction *P* values were corrected for the three comparisons using the Holm method. The Holm method was selected to control the family-wise error rate across the three interaction tests. For potential effect modifiers showing evidence of interaction, stratum-specific RCS analyses were planned to further explore the shape of the continuous calculated RC–GDM association, with the variable defining the stratum omitted from the corresponding adjustment model.

Furthermore, after dividing the study population into two groups based on the exploratory data-derived calculated RC cut-off, we compared the incidence of GDM and other obstetric outcomes (including hypertensive disorders of pregnancy [HDP], prelabor rupture of membranes [PROM], gestational age at delivery, and preterm birth [PTB]) between the two groups. Categorical variables were compared using the chi-square test or Fisher’s exact test, while continuous variables were compared using the independent-samples Student’s t-test or Mann–Whitney U test.

The primary multivariable logistic regression model was assessed for the functional form of continuous covariates, influential observations, and overall goodness of fit. Potentially influential observations were evaluated using Cook’s distance, and model fit was assessed using the Hosmer–Lemeshow test.

Several sensitivity analyses were performed to examine the influence of extreme calculated RC values and the potential collinearity between calculated RC and TG. First, because of the potential collinearity between calculated RC and TG, multivariable logistic regression models were re-fitted with TG replacing calculated RC and with both calculated RC and TG entered simultaneously as candidate exposures. Collinearity and coefficient stability were assessed using VIFs. The mutually adjusted model was used to evaluate coefficient stability in the presence of collinearity and was not interpreted as estimating independent effects of calculated RC and TG. Second, because calculated RC had a markedly right-skewed distribution, a percentile-based sensitivity analysis was performed. Calculated RC values above the 99th percentile were winsorized to the 99th-percentile value, and the primary adjusted continuous-exposure logistic regression model was repeated using the winsorized variable. The percentile threshold was calculated from the calculated RC distribution in the full analytic cohort. This sensitivity-analysis model was adjusted for the same covariates as the primary continuous-exposure model.

## 3 Results

### 3.1 Baseline characteristics of the study population

**Figure 1:**
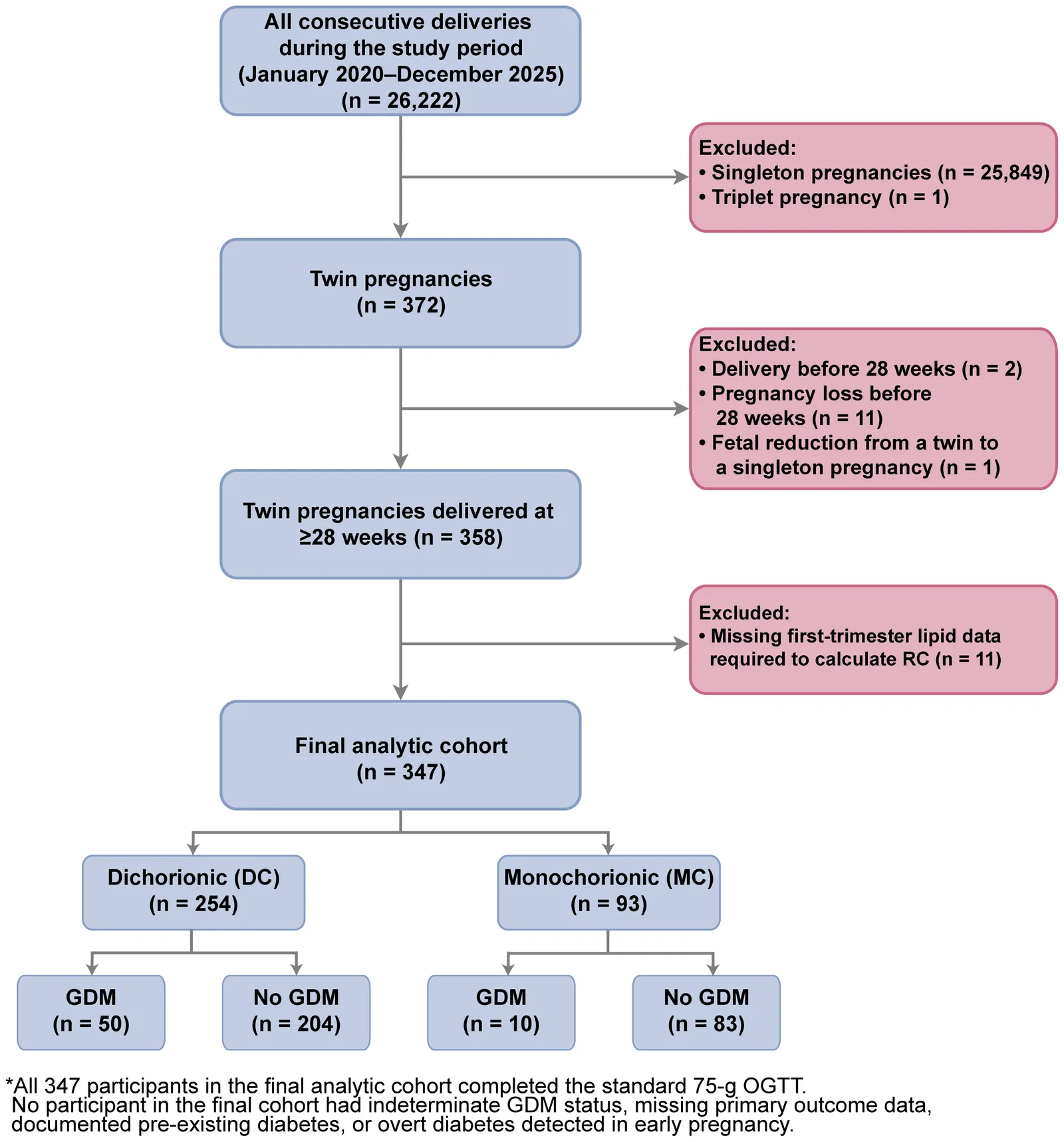
Flow diagram of participant selection. The final analytic cohort comprised 347 women with twin pregnancies, including 254 dichorionic and 93 monochorionic pregnancies. All 347 participants in the final analytic cohort completed the standard 75-g OGTT. No participant in the final cohort had indeterminate GDM status, missing primary outcome data, documented pre-existing diabetes, or overt diabetes detected in early pregnancy. RC, remnant cholesterol; GDM, gestational diabetes mellitus; DC, dichorionic; MC, monochorionic.

Among 26,222 consecutive deliveries during the study period, 25,849 singleton pregnancies and one triplet pregnancy were excluded, leaving 372 twin pregnancies. A further 14 pregnancies were excluded because of delivery before 28 weeks, pregnancy loss, or fetal reduction, and 11 were excluded because of missing first-trimester lipid data required to calculate RC. Review of the medical records identified no participant with documented pre-existing diabetes or overt diabetes detected in early pregnancy. All 347 participants included in the final analytic cohort had complete OGTT data, and no participant had indeterminate GDM status. Finally, a total of 347 women with twin pregnancies were enrolled (**Figure 1**), of whom 60 (17.3%) were diagnosed with GDM and 287 (82.7%) did not develop GDM. Baseline characteristics of the study population and comparisons between the GDM and non-GDM groups are detailed in **Table 1**. Compared with the no-GDM group, women in the GDM group were older (32.00 [28.00–36.00] vs. 30.00 [27.00–32.00] years, P < 0.001), had higher pre-pregnancy BMI (21.63 [19.77–24.07] vs. 20.70 [19.10–22.63] kg/m², P = 0.035), and were more likely to have undergone ART (53.3% vs. 38.7%, P = 0.036). Additionally, TG levels were significantly higher in the GDM group (4.54 [3.48–5.61] vs. 3.96 [3.11–4.95] mmol/L, P = 0.043), and calculated RC levels were also significantly higher in the GDM group (1.60 [1.18–2.25] vs. 1.21 [0.83–1.69] mmol/L, P < 0.001). Chorionicity did not differ significantly between the groups (P = 0.051), although the proportion of dichorionic twins was numerically higher in the GDM group (83.3% vs. 71.1%). Education level, gravidity, primiparous status, TC, HDL-C, and LDL-C were not significantly different between the two groups. Calculated RC levels did not differ significantly between dichorionic and monochorionic pregnancies (median [IQR] 1.35 [0.90–1.87] vs. 1.11 [0.77–1.67] mmol/L, P = 0.063). The complete lipid distributions and observed ranges are presented in **Supplementary Table 1**. The maximum TG and calculated RC concentrations were verified against the source laboratory records, and no data-entry, decimal-point, or unit-conversion errors were identified. Independently recomputed calculated RC values were consistent with their recorded lipid components. No negative calculated RC values occurred, and no values were truncated or set to zero. Three participants had a calculated RC concentration of exactly 0.00 mmol/L because TC equaled the sum of directly measured LDL-C and HDL-C at the precision reported by the laboratory.

**Table 1:** Characteristics of the study participants.

Variables	Total (n = 347)	No GDM (n = 287)	GDM (n = 60)	*P* value
Age, years, median (IQR)	30.00 (27.00–33.00)	30.00 (27.00–32.00)	32.00 (28.00–36.00)	<0.001
Pre-pregnancy BMI, kg/m², median (IQR)	20.90 (19.14–22.86)	20.70 (19.10–22.63)	21.63 (19.77–24.07)	0.035
Education, n (%)				0.289
Junior high school and below	72 (20.7)	56 (19.5)	16 (26.7)	
Senior high school/technical secondary school/vocational high school	73 (21.0)	64 (22.3)	9 (15.0)	
College or above	202 (58.2)	167 (58.2)	35 (58.3)	
Gravidity, median (IQR)	2.00 (1.00–3.00)	2.00 (1.00–3.00)	2.00 (1.00–3.00)	0.433
Primiparous, n (%)	223 (64.3)	187 (65.2)	36 (60.0)	0.448
ART, n (%)	143 (41.2)	111 (38.7)	32 (53.3)	0.036
Chorionicity, n (%)				0.051
Dichorionic	254 (73.2)	204 (71.1)	50 (83.3)	
Monochorionic	93 (26.8)	83 (28.9)	10 (16.7)	
TC, mmol/L, median (IQR)	6.55 (5.76–7.22)	6.58 (5.71–7.17)	6.49 (5.93–7.59)	0.307
HDL-C, mmol/L, median (IQR)	1.43 (1.23–1.68)	1.42 (1.23–1.69)	1.44 (1.20–1.65)	0.758
LDL-C, mmol/L, median (IQR)	3.65 (2.80–4.28)	3.70 (2.84–4.28)	3.37 (2.51–4.14)	0.156
TG, mmol/L, median (IQR)	4.04 (3.18–5.07)	3.96 (3.11–4.95)	4.54 (3.48–5.61)	0.043
Calculated RC, mmol/L, median (IQR)	1.28 (0.87–1.86)	1.21 (0.83–1.69)	1.60 (1.18–2.25)	<0.001

GDM, gestational diabetes mellitus; BMI, body mass index; ART, assisted reproductive technology; TC, total cholesterol; HDL-C, high-density lipoprotein cholesterol; LDL-C, low-density lipoprotein cholesterol; TG, triglycerides; RC, remnant cholesterol; IQR, interquartile range.

### 3.2 Dose–response relationship between calculated RC and GDM risk

**Figure 2:**
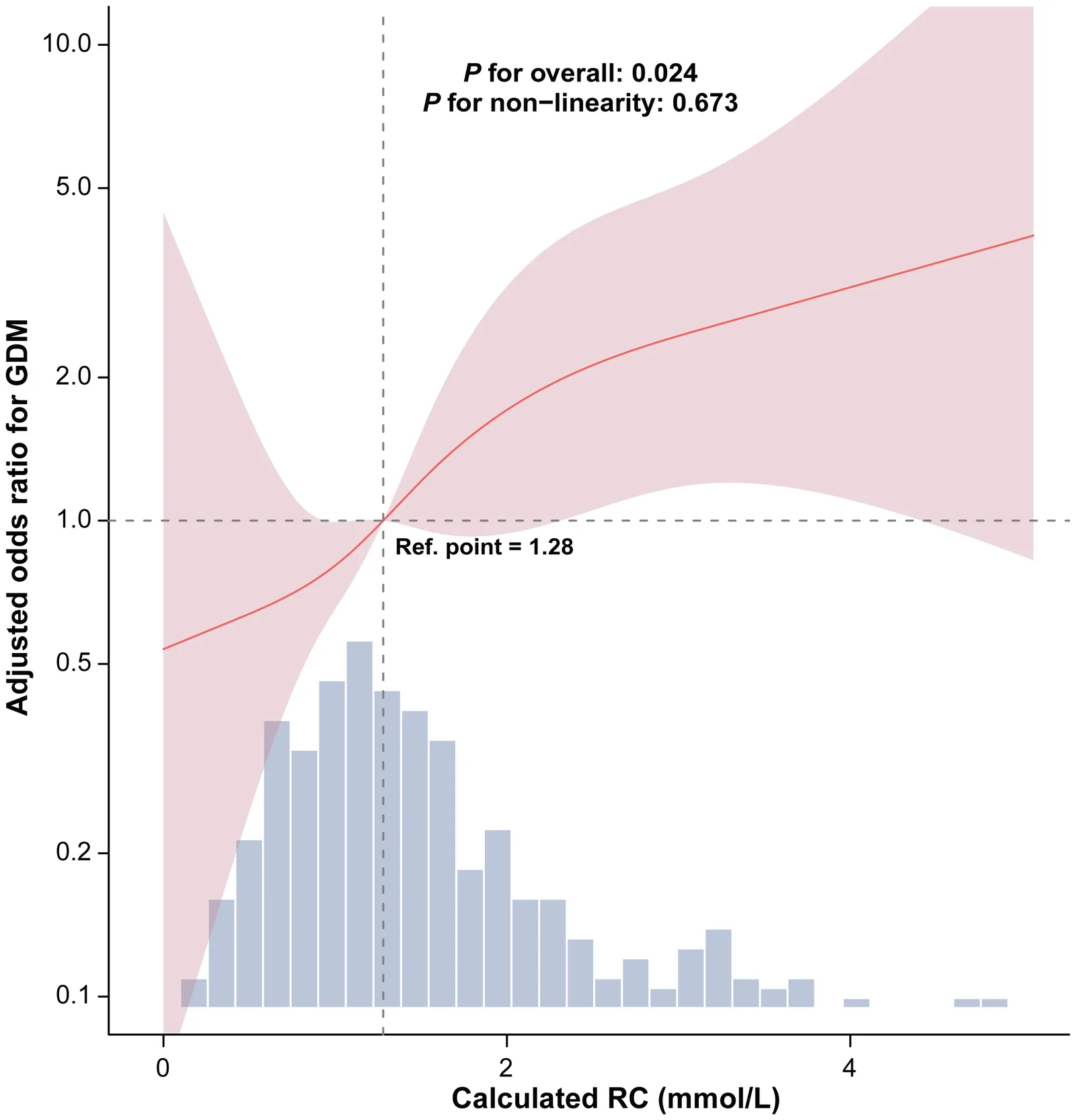
RCS analysis of the association between first-trimester calculated RC and GDM in women with twin pregnancies. The red solid line represents the adjusted odds ratio (OR), and the light-red shaded area represents the 95% confidence interval. OR values are displayed on a logarithmic vertical scale. The horizontal dashed line indicates OR = 1.00, and the vertical dashed line indicates the median calculated RC concentration of 1.28 mmol/L, which was used as the reference value (OR = 1.00). The light-blue background histogram shows the distribution of calculated RC in the full analytic cohort. All participants were included in model estimation and hypothesis testing. The model was adjusted for age, education, pre-pregnancy BMI, assisted reproductive technology, gravidity, primiparous status, and chorionicity. RCS, restricted cubic spline; RC, remnant cholesterol; GDM, gestational diabetes mellitus; OR, odds ratio; CI, confidence interval; BMI, body mass index; ART, assisted reproductive technology.

After adjustment for age, education, pre-pregnancy BMI, ART, gravidity, primiparous status, and chorionicity, the restricted cubic spline analysis showed an overall association between calculated RC and GDM (*P* for overall=0.024), with no evidence of non-linearity (*P* for non-linearity=0.673) (**Figure 2**). These findings were consistent with modeling calculated RC as a continuous linear term in the primary logistic regression analysis. The background histogram represents the distribution of calculated RC in the study population, the solid line represents the adjusted odds ratio, and the shaded area represents the 95% CI.

### 3.3 Multivariable logistic regression analysis of the association between calculated RC and GDM

In the primary continuous-exposure analysis, each 1 mmol/L increase in calculated RC was associated with higher odds of GDM in the unadjusted model (OR 1.71, 95% CI 1.27–2.30; P < 0.001) and after covariate adjustment (adjusted OR 1.63, 95% CI 1.17–2.26; P = 0.004). No single highly influential observation was identified based on Cook’s distance, and the Hosmer–Lemeshow test showed no evidence of poor model fit (χ²=6.33, df=8; P = 0.610).

In the secondary exploratory analysis of the stand-alone discrimination of calculated RC, the data-derived Youden-index cut-off was 1.145 mmol/L (**Supplementary Figure 1**). At this cut-off, sensitivity was 78.33% (95% CI 66.4–86.9%), specificity was 44.95% (95% CI 39.3–50.7%), positive predictive value was 22.93% (95% CI 17.7–29.1%), negative predictive value was 90.85% (95% CI 85.0–94.6%), and the AUC was 0.649 (95% CI 0.572–0.724), with a Youden index of 0.233. Because this exploratory cut-off was derived and evaluated in the same cohort, these estimates describe in-sample stand-alone discrimination and do not establish incremental predictive value or clinical utility. The NPV should be interpreted in the context of the 17.3% GDM prevalence in this cohort and should not be considered evidence that calculated RC can rule out GDM.

After converting calculated RC into a categorical variable using this cut-off, calculated RC ≥1.145 mmol/L was associated with higher odds of GDM in the unadjusted analysis (OR 2.95, 95% CI 1.53–5.69; P = 0.001). After adjustment for age, education, pre-pregnancy BMI, ART, gravidity, primiparous status, and chorionicity, the association remained statistically significant (adjusted OR 2.56, 95% CI 1.28–5.10; P = 0.008) (**Table 2**).

**Table 2:** Associations of continuous and dichotomized calculated RC with GDM in the full cohort.

Variables	Total, n	GDM events, n (%)	Crude OR (95% CI)	*P* value	Adjusted OR (95% CI)	*P* value
Calculated RC (mmol/L)	347	60 (17.3)	1.71 (1.27–2.30)	<0.001	1.63 (1.17–2.26)	0.004
Calculated RC <1.145 mmol/L	142	13 (9.2)	1.00 (Ref)	–	1.00 (Ref)	–
Calculated RC ≥1.145 mmol/L	205	47 (22.9)	2.95 (1.53–5.69)	0.001	2.56 (1.28–5.10)	0.008

The adjusted models included age, education, pre-pregnancy BMI, ART, gravidity, primiparous status, and chorionicity. Education was modeled as a three-level categorical variable, and gravidity was modeled as a continuous variable. The continuous calculated RC estimate represents the change in the odds of GDM per 1 mmol/L increase. For the dichotomized analysis, calculated RC <1.145 mmol/L was the reference category, and the reported OR compares calculated RC ≥1.145 mmol/L with calculated RC <1.145 mmol/L in the full cohort. RC, remnant cholesterol; GDM, gestational diabetes mellitus; BMI, body mass index; ART, assisted reproductive technology; OR, odds ratio; CI, confidence interval.

### 3.4 Subgroup analysis and interaction analysis

**Figure 3:**
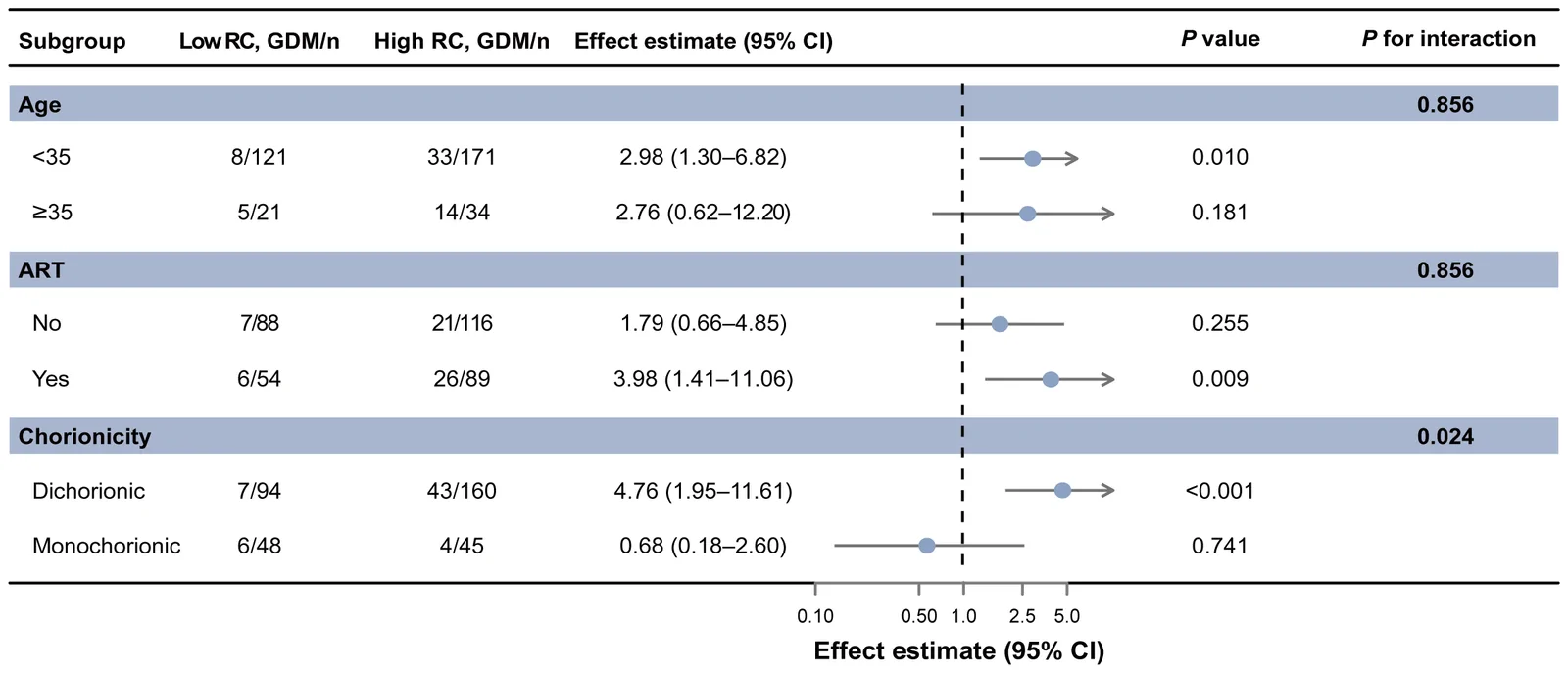
Subgroup analyses of the association between dichotomized calculated RC and GDM. Within each age, ART, and chorionicity subgroup, calculated RC <1.145 mmol/L was the reference category, and the effect estimate compares calculated RC ≥1.145 mmol/L with calculated RC <1.145 mmol/L. The columns “Low RC, GDM/n” and “High RC, GDM/n” report the number of GDM events and the total number of participants in each calculated RC category. Adjusted ORs were obtained from separate multivariable logistic regression models fitted within each subgroup, with the variable defining the subgroup omitted from the corresponding adjustment set. Because only 10 GDM events occurred in the monochorionic subgroup, the estimate for this subgroup is the crude OR, and its *P* value was obtained using Fisher’s exact test. Interaction *P* values were obtained from full-cohort models containing the corresponding cross-product terms and were adjusted across the three interaction tests using the Holm method. RC, remnant cholesterol; GDM, gestational diabetes mellitus; ART, assisted reproductive technology; OR, odds ratio; CI, confidence interval.

To explore potential heterogeneity in the calculated RC–GDM association, subgroup analyses were conducted according to age, ART, and chorionicity after the study population was divided into the calculated RC <1.145 mmol/L group (n = 142) and the calculated RC ≥1.145 mmol/L group (n = 205). Within each subgroup, the reported OR compared calculated RC ≥1.145 mmol/L with calculated RC <1.145 mmol/L, with the latter serving as the reference category. The sample size and GDM event count for both calculated RC groups within each subgroup are reported in **Figure 3**. Interaction tests were performed using full-cohort models adjusted for the prespecified covariates, and the three interaction P values were adjusted using the Holm method. An exploratory interaction by chorionicity was observed in the association between calculated RC and GDM (interaction coefficient β=2.19, 95% CI 0.54–3.83; raw likelihood ratio test P = 0.008; Holm-adjusted P for interaction=0.024), although the wide confidence interval and the limited number of GDM events in the monochorionic subgroup indicated substantial uncertainty in the magnitude and stability of the interaction. In contrast, no significant interaction was observed for age (P for interaction=0.856) or ART (P for interaction=0.856). Within the dichorionic subgroup, GDM occurred in 7.4% (7/94) of women with calculated RC <1.145 mmol/L and 26.9% (43/160) of women with calculated RC ≥1.145 mmol/L; the adjusted within-stratum OR was 4.76 (95% CI 1.95–11.61; P < 0.001). Within the monochorionic subgroup, GDM occurred in 12.5% (6/48) of women with calculated RC <1.145 mmol/L and 8.9% (4/45) of women with calculated RC ≥1.145 mmol/L. Because only 10 GDM events occurred in this subgroup, a multivariable within-stratum estimate was not reported. The crude OR comparing calculated RC ≥1.145 mmol/L with calculated RC <1.145 mmol/L was 0.68 (95% CI 0.18–2.60; Fisher’s exact P = 0.741).

**Figure 4:**
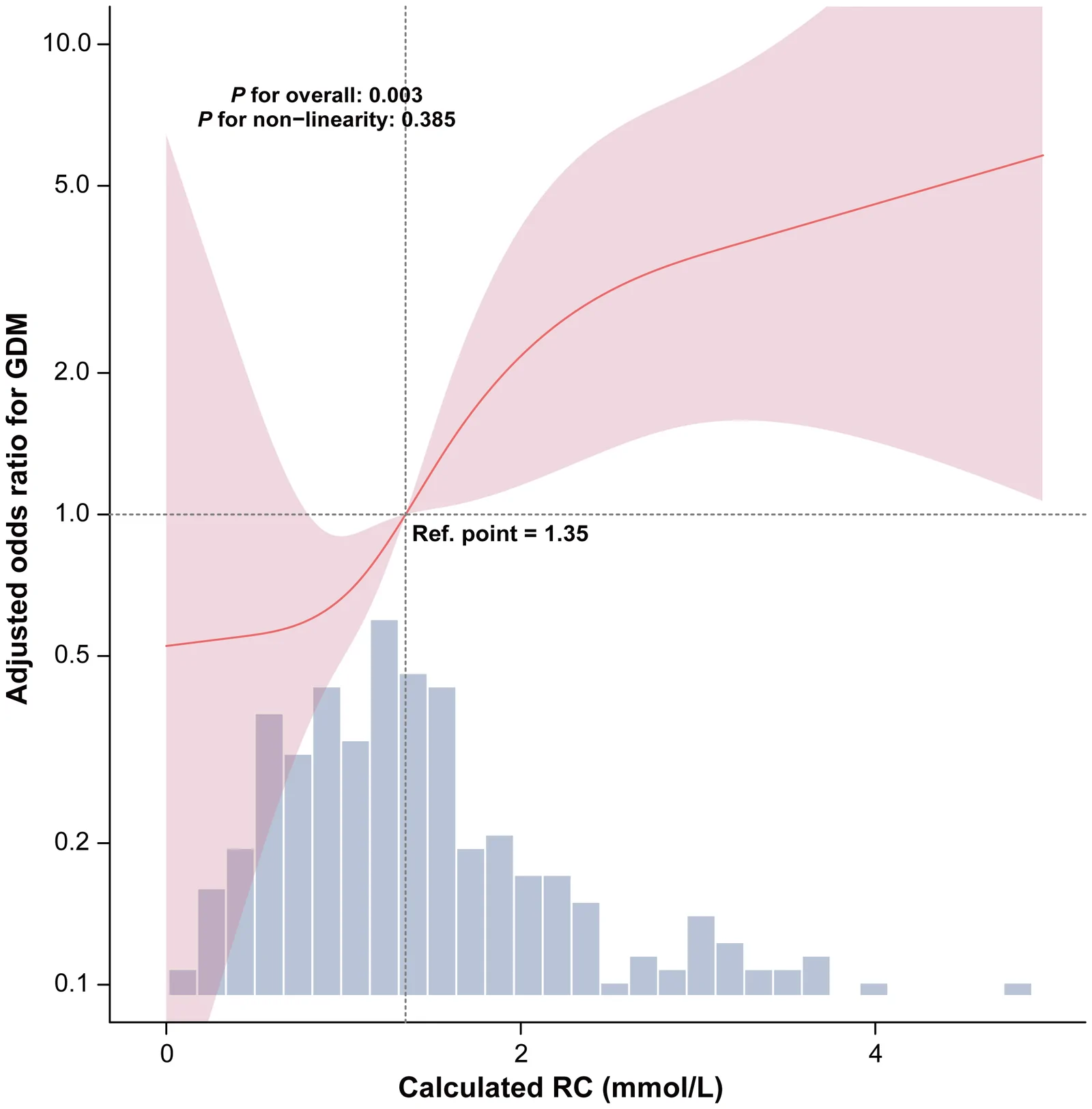
Exploratory restricted cubic spline analysis of the association between first-trimester calculated remnant cholesterol and gestational diabetes mellitus in dichorionic twin pregnancies. The red solid line represents the adjusted odds ratio (OR), and the light-red shaded area represents the 95% confidence interval (CI). OR values are displayed on a logarithmic vertical scale. The horizontal dashed line indicates OR = 1.00, and the vertical dashed line indicates the median calculated RC concentration of 1.35 mmol/L, which was used as the reference value (OR = 1.00). The light-blue background histogram shows the distribution of calculated RC in the dichorionic subgroup. The model was adjusted for age, education, pre-pregnancy BMI, assisted reproductive technology, gravidity, and primiparous status. This analysis included 254 dichorionic twin pregnancies and was exploratory. RCS, restricted cubic spline; RC, remnant cholesterol; GDM, gestational diabetes mellitus; OR, odds ratio; CI, confidence interval; BMI, body mass index.

Given the observed interaction by chorionicity, stratum-specific RCS analysis was considered. Because only 10 GDM events occurred in the monochorionic subgroup, the available data were insufficient to estimate the continuous dose–response relationship reliably. Therefore, an exploratory RCS analysis was conducted only in the dichorionic subgroup and showed an overall association (P for overall=0.003), with no evidence of non-linearity (P for non-linearity=0.385) (**Figure 4**).

### 3.5 Comparisons of clinical outcomes stratified by RC threshold

As shown in **Table 3**, the incidence of GDM was significantly higher in the calculated RC ≥1.145 mmol/L group than in the calculated RC <1.145 mmol/L group (22.9% vs. 9.2%, P < 0.001). However, no significant differences were observed between the two groups in hypertensive disorders of pregnancy (HDP), prelabor rupture of membranes (PROM), gestational age at delivery, or preterm birth (PTB) (P = 0.883, P = 0.475, P = 0.469, and P = 0.640, respectively).

**Table 3:** Comparisons of clinical outcomes stratified by the calculated RC threshold.

Variables	Total (n = 347)	Calculated RC <1.145 mmol/L (n = 142)	Calculated RC ≥1.145 mmol/L (n = 205)	*P* value
GDM, n (%)	60 (17.3)	13 (9.2)	47 (22.9)	<0.001
HDP, n (%)	106 (30.5)	44 (31.0)	62 (30.2)	0.883
PROM, n (%)	67 (19.3)	30 (21.1)	37 (18.0)	0.475
Gestational age at delivery, weeks, Mean ± SD	35.9 ± 1.7	35.8 ± 1.8	36.0 ± 1.6	0.469
PTB, n (%)	242 (69.7)	101 (71.1)	141 (68.8)	0.640

RC, remnant cholesterol; GDM, gestational diabetes mellitus; HDP, hypertensive disorders of pregnancy; PROM, prelabor rupture of membranes; PTB, preterm birth; SD, standard deviation.

### 3.6 Sensitivity analysis

In the percentile-based sensitivity analysis, the 99th percentile of calculated RC was 3.968 mmol/L. Values above this threshold were winsorized to 3.968 mmol/L, affecting four participants. After adjustment for age, education, pre-pregnancy BMI, ART, gravidity, primiparous status, and chorionicity, continuous calculated RC remained associated with GDM (adjusted OR 1.66, 95% CI 1.17–2.35; P = 0.004). This estimate was consistent with the primary analysis using the original calculated RC values (adjusted OR 1.63, 95% CI 1.17–2.26; P = 0.004) (**Supplementary Table 2**).

When modeled separately, the adjusted ORs were 1.63 (95% CI 1.17–2.26; P = 0.004) for calculated RC and 1.10 (95% CI 0.97–1.25; P = 0.121) for TG. When calculated RC and TG were included simultaneously, the adjusted ORs were 3.97 (95% CI 1.77–8.89; P < 0.001) and 0.68 (95% CI 0.49–0.93; P = 0.016), respectively. The corresponding VIF values for calculated RC and TG were approximately 6, whereas the VIFs for the other covariates were below 2.4 (**Supplementary Table 3**). The marked coefficient changes and reversal in the direction of the TG estimate indicated substantial collinearity, coefficient instability, and possible suppression. Therefore, the mutually adjusted model was treated only as a diagnostic of collinearity and coefficient stability, and its OR estimates were not interpreted biologically as independent effects of calculated RC or TG or as evidence that calculated RC was superior to TG (**Supplementary Table 4**).

## 4 Discussion

Based on a twin pregnancy cohort from a Chinese tertiary hospital, this study systematically evaluated the association between first-trimester RC and GDM, and explored potential effect modification by chorionicity. After adjustment for age, education, pre-pregnancy BMI, ART, gravidity, primiparous status, and chorionicity, calculated RC was associated with higher odds of GDM, with no evidence of non-linearity. The internally derived calculated RC cut-off was 1.145 mmol/L, and women with calculated RC ≥1.145 mmol/L had 2.56-fold higher odds of GDM than those with calculated RC <1.145 mmol/L. An exploratory interaction by chorionicity was observed, although the wide confidence interval and the limited number of GDM events in the monochorionic subgroup indicated substantial uncertainty in its magnitude and stability. No statistically significant associations were observed between the calculated RC cut-off and the other examined obstetric outcomes; however, these null findings should not be interpreted as evidence that the association is specific to GDM. Because calculated RC and TG showed substantial collinearity and coefficient instability when entered simultaneously, the mutually adjusted estimates were treated only as a collinearity diagnostic and should not be interpreted as biologically independent effects. Collectively, these findings support further evaluation of calculated RC as an early-pregnancy lipid measure associated with subsequent GDM in women with twin pregnancies.

Prior studies have explored the association between RC and metabolic diseases, but have predominantly focused on cardiovascular outcomes in non-pregnant populations (8 9). Varbo et al. (8), based on the Copenhagen General Population Study and the Copenhagen City Heart Study, demonstrated that RC is an independent causal risk factor for ischemic heart disease; however, the study outcomes were limited to non-pregnant populations and did not extend to gestational metabolic outcomes. Zhao et al. (9) further reported that higher RC levels were associated with an increased risk of ischemic heart disease in a prospective cohort of approximately 42,000 participants from the general population, with a median follow-up of nearly 10 years. However, this study focused on middle-aged and older adults and did not evaluate RC in the metabolic context of pregnancy. Gao et al. (12) reported a positive association between calculated RC and GDM in a study of 590 women with singleton pregnancies; however, the study did not include women with twin pregnancies. Su et al. (25) further confirmed the association between elevated first-trimester RC and increased GDM risk in a Chinese singleton pregnancy cohort, but the study design was likewise limited to singleton pregnancies and did not evaluate the association between calculated RC and GDM in twin pregnancies. The present study extends previous observations from singleton to twin pregnancies, a population with distinct metabolic and placental characteristics. The RCS analysis allowed the shape of the calculated RC–GDM association to be examined without imposing a simple categorical structure.

The observed association between first-trimester calculated RC and subsequent GDM can be considered within the broader biological framework linking triglyceride-rich lipoprotein metabolism to impaired glucose regulation. Previous studies have suggested that remnant lipoproteins may be related to chronic low-grade inflammation (23), insulin resistance (26, 27), lipotoxicity and pancreatic β-cell dysfunction (28–30), and endothelial dysfunction (11, 31, 32). These pathways provide biologically plausible hypotheses for understanding why elevated calculated RC in early pregnancy may be associated with subsequent GDM and support further investigation of calculated RC as a marker of early metabolic disturbance. However, the present study did not directly measure insulin sensitivity, inflammatory biomarkers, lipoprotein-particle composition, pancreatic β-cell function, or endothelial function. Therefore, these pathways were not evaluated in this cohort and should not be interpreted as mechanisms established by the present findings. Prospective studies incorporating direct assessments of insulin resistance, inflammatory and lipidomic profiles, and β-cell function are needed to determine whether any of these pathways mediate the calculated RC–GDM association. Thus, the present results provide observational and hypothesis-generating evidence for future mechanistic research but do not establish causality or any specific biological pathway.

An exploratory interaction by chorionicity was observed in the calculated RC–GDM association. However, the monochorionic subgroup included only 10 GDM events, and its within-stratum estimate was imprecise. The interaction analysis was also based on the internally derived calculated RC cut-off of 1.145 mmol/L, making the result potentially sample-dependent. The small number of subgroup events, model instability, and use of a data-derived cut-off limit the stability and reproducibility of this finding. Therefore, the interaction should be regarded as hypothesis-generating rather than as definitive evidence of effect modification. Although dichorionic and monochorionic pregnancies differ in placental organization and fetal circulatory relationships, the present study did not measure placental hormones, placental pathology, vascular anastomoses, or other placental functional characteristics. Accordingly, no mechanistic role of placental structure can be inferred from the observed subgroup pattern. Further exploration of potential heterogeneity in the calculated RC–GDM association by chorionicity is warranted in large prospective multicenter cohorts with adequate numbers of monochorionic twin pregnancies and GDM events.

The incidence of GDM was significantly higher among women with calculated RC ≥1.145 mmol/L than among those with calculated RC <1.145 mmol/L, whereas no statistically significant differences were observed for HDP, PROM, gestational age at delivery, or PTB. However, the absence of statistically significant associations with these secondary outcomes does not establish that calculated RC is specific to GDM. These secondary analyses were exploratory and may have had insufficient statistical power to detect clinically relevant associations with other obstetric outcomes.

First-trimester calculated RC can be derived from a standard fasting lipid panel without requiring an additional assay, making it accessible for further etiological and metabolic research. The exploratory ROC analysis evaluated only the apparent in-sample discrimination of calculated RC as a stand-alone measure and did not assess whether calculated RC improves prediction beyond established clinical risk factors. Its stand-alone discrimination was modest, and the internally derived cut-off requires external validation. In particular, the observed NPV is prevalence-dependent and does not establish a safe rule-out strategy. The present findings therefore do not support the use of calculated RC for clinical risk stratification, alteration of routine glucose monitoring, initiation of early interventions, or replacement of the standard 75-g OGTT at 24–28 weeks. Regardless of the calculated RC level, all women should continue to undergo standard guideline-recommended GDM screening.

This study has several limitations that warrant consideration. First, the single-center, retrospective design may limit generalizability and introduce selection bias. Because the source population was restricted to women who delivered at our hospital, the cohort did not represent all twin pregnancies identified during early pregnancy. Pregnancies involving pregnancy loss, delivery before 28 weeks, fetal reduction, or transfer to another institution before delivery were not included. Therefore, selection related to pregnancy outcomes, referral and delivery patterns, or severe complications cannot be excluded. The limited information available for women excluded because of missing lipid data also prevented a comprehensive assessment of selection bias. Multicenter studies are needed to assess the generalizability of our findings. Second, the monochorionic subgroup included only 93 women and 10 GDM events, resulting in an imprecise estimate and limited stability of the interaction model. Moreover, the interaction analysis used an internally derived calculated RC cut-off, which may have increased its sample dependence. Therefore, the observed interaction by chorionicity should be regarded as a hypothesis-generating finding and should be further explored in large prospective multicenter cohorts with adequate numbers of monochorionic twin pregnancies and GDM events. Third, although the models were adjusted for age, education, pre-pregnancy BMI, ART, gravidity, primiparous status, and chorionicity, residual confounding cannot be excluded. Potential confounders, including family history of diabetes, dietary patterns, physical activity, gestational weight gain, and additional early-pregnancy glycemic markers, were not consistently available in the retrospective medical records. These unmeasured factors may partly explain the observed association between calculated RC and GDM. Therefore, the present observational findings do not allow us to determine whether calculated RC has an independent causal effect on GDM risk. In addition, insulin resistance, inflammatory pathways, lipoprotein-particle composition, pancreatic β-cell function, placental hormones, placental pathology, and vascular anastomoses were not measured. Therefore, the biological mechanisms underlying the observed association and its potential heterogeneity by chorionicity could not be evaluated. Fourth, calculated RC was derived from a standard lipid panel (TC − [LDL-C + HDL-C]) rather than directly measured by ultracentrifugation or nuclear magnetic resonance spectroscopy; this non-differential misclassification would be expected to bias the observed association toward the null. Information bias from routine clinical assays and self-reported pre-pregnancy BMI is also likely non-differential between GDM and non-GDM groups. Fifth, the exploratory calculated RC cut-off was derived and evaluated within the same cohort and may therefore be sample-dependent. The analysis assessed calculated RC as a stand-alone marker. The bootstrap procedure provided a percentile confidence interval for the apparent AUC but did not provide optimism correction or out-of-bag validation. We did not construct a multivariable clinical prediction model or evaluate the incremental contribution of calculated RC beyond established clinical variables. Therefore, the reported AUC, NPV, and cut-off should not be interpreted as evidence of stable predictive performance, incremental predictive value, or clinical utility. Future research may explore the longitudinal trajectory of calculated RC across pregnancy and its temporal relationship with GDM onset, further investigate potential heterogeneity by chorionicity in large prospective multicenter cohorts, evaluate the incremental and externally validated discriminative performance of calculated RC in appropriately designed prediction studies in independent cohorts, and integrate lipidomics, inflammatory profiles, and placental pathology to elucidate the potential pathways underlying the calculated RC–GDM association. Whether interventions targeting remnant-lipoprotein metabolism can influence GDM risk requires investigation in appropriately designed prospective studies.

## 5 Conclusion

In this retrospective cohort of women with twin pregnancies, elevated first-trimester calculated RC was associated with higher odds of GDM. Its stand-alone discrimination was modest, and the internally derived 1.145 mmol/L cut-off should be regarded as exploratory rather than as an established clinical threshold. The potential heterogeneity by chorionicity observed in this study should be regarded as hypothesis-generating and further explored in large prospective multicenter cohorts with adequate numbers of monochorionic twin pregnancies and GDM events. These findings support further investigation of calculated RC as an early-pregnancy lipid measure associated with GDM, including evaluation of whether it provides additional information beyond established clinical factors.

## Data Availability

The raw data supporting the conclusions of this article will be made available by the authors, without undue reservation.
