# Supplementary material for: First-trimester calculated remnant cholesterol and gestational diabetes mellitus in twin pregnancies: a retrospective cohort study
Source: Front Endocrinol (Lausanne). 2026 Sep 11;17:1882821. doi: 10.3389/fendo.2026.1882821 (PMC13612319; doi:10.3389/fendo.2026.1882821)
Supplement: Supplementary file 1 [file SupplementaryFile1.docx]

**Supplementary Figure 1. Receiver operating characteristic curve describing the stand-alone discrimination of first-trimester calculated RC for GDM.** The stair-step curve represents the ROC curve, and the red dot indicates the exploratory data-derived cut-off selected by maximizing the Youden index. Abbreviations: RC, remnant cholesterol; GDM, gestational diabetes mellitus; AUC, area under the curve; CI, confidence interval; ROC, receiver operating characteristic.
