# Supplementary material for: First-trimester calculated remnant cholesterol and gestational diabetes mellitus in twin pregnancies: a retrospective cohort study
Source: Front Endocrinol (Lausanne). 2026 Sep 11;17:1882821. doi: 10.3389/fendo.2026.1882821 (PMC13612319; doi:10.3389/fendo.2026.1882821)
Supplement: Supplementary file 2 [file SupplementaryFile2.docx]

**Supplementary Table 1. Distribution and observed ranges of first-trimester lipid measurements.**

| **Lipid measure** | **n** | **Median (IQR), mmol/L** | **Minimum, mmol/L** | **Maximum, mmol/L** |
| --- | --- | --- | --- | --- |
| TC | 347 | 6.55 (5.76–7.22) | 3.44 | 10.84 |
| HDL-C | 347 | 1.43 (1.23–1.68) | 0.42 | 2.79 |
| Directly measured LDL-C | 347 | 3.65 (2.80–4.28) | 1.13 | 8.71 |
| TG | 347 | 4.04 (3.18–5.07) | 1.53 | 20.34 |
| Calculated RC | 347 | 1.28 (0.87–1.86) | 0.00 | 7.97 |

All measurements were obtained at 10–14 weeks of gestation from the same early-morning blood sample after participants reported being fasting. The exact fasting duration was not consistently documented. Calculated RC was derived as TC − LDL-C − HDL-C using the laboratory-reported values, which were reported to two decimal places. No additional rounding was applied by the investigators before calculation. Three participants had a calculated RC value of 0.00 mmol/L. No negative calculated RC values occurred, and no values were truncated or set to zero. Abbreviations: TC, total cholesterol; HDL-C, high-density lipoprotein cholesterol; LDL-C, low-density lipoprotein cholesterol; TG, triglycerides; RC, remnant cholesterol; IQR, interquartile range.

**Supplementary Table 2. Sensitivity analysis of the association between calculated remnant cholesterol and gestational diabetes mellitus after winsorization at the 99th percentile**

| **Analysis** | **Calculated RC treatment** | **Adjusted OR (95% CI)** | ***P* value** |
| --- | --- | --- | --- |
| Primary analysis | Original calculated RC, per 1 mmol/L increase | 1.63 (1.17–2.26) | 0.004 |
| Sensitivity analysis | Calculated RC winsorized at the 99th percentile, per 1 mmol/L increase | 1.66 (1.17–2.35) | 0.004 |

The 99th percentile of calculated RC was 3.968 mmol/L. Values above this threshold were winsorized to 3.968 mmol/L, affecting four participants. Both models were adjusted for age, education, pre-pregnancy body mass index, assisted reproductive technology, gravidity, primiparous status, and chorionicity. The primary analysis used the original calculated RC values, whereas the sensitivity analysis used calculated RC after 99th-percentile winsorization. Abbreviations: RC, remnant cholesterol; GDM, gestational diabetes mellitus; OR, odds ratio; CI, confidence interval.

**Supplementary Table 3.** **Variance Inflation Factors of Covariates.**

| **Variables** | **VIF** |
| --- | --- |
| Calculated RC | **6.011** |
| Age | 1.354 |
| Education | 1.280 |
| ART | 1.434 |
| Gravidity | 2.232 |
| Primiparous status | 2.335 |
| Pre-pregnancy BMI | 1.130 |
| Chorionicity | 1.191 |
| TG | **6.072** |

Variance inflation factors were calculated from the mutually adjusted model containing continuous calculated RC, continuous TG, and the prespecified covariates. Abbreviations: VIF, variance inflation factor; RC, remnant cholesterol; TG, triglycerides; BMI, body mass index; ART, assisted reproductive technology.

**Supplementary Table 4. Separate and mutually adjusted associations of calculated RC and triglycerides with GDM.**

| **Model** | **Exposure** | **Adjusted OR (95% CI)** | ***P* value** | **VIF** |
| --- | --- | --- | --- | --- |
| Separate model | Calculated RC, per 1 mmol/L | 1.63 (1.17–2.26) | 0.004 | — |
| Separate model | TG, per 1 mmol/L | 1.10 (0.97–1.25) | 0.121 | — |
| Mutually adjusted model | Calculated RC, per 1 mmol/L | 3.97 (1.77–8.89) | <0.001 | 6.011 |
| Mutually adjusted model | TG, per 1 mmol/L | 0.68 (0.49–0.93) | 0.016 | 6.072 |

All models were adjusted for age, education, pre-pregnancy BMI, ART, gravidity, primiparous status, and chorionicity. Calculated RC and TG were modeled as continuous exposures, and the reported ORs represent the change in the odds of GDM per 1 mmol/L increase. The mutually adjusted model was fitted only to assess collinearity and coefficient stability. Because of the substantial collinearity between calculated RC and TG, its OR estimates should not be interpreted as biologically independent effects of calculated RC or TG. Abbreviations: RC, remnant cholesterol; TG, triglycerides; GDM, gestational diabetes mellitus; OR, odds ratio; CI, confidence interval; VIF, variance inflation factor; BMI, body mass index; ART, assisted reproductive technology.
